# Observation of wet specimens sensitive to evaporation using scanning electron microscopy

**DOI:** 10.1093/jmicro/dfy041

**Published:** 2018-10-11

**Authors:** Noriyuki Inoue, Yoshiko Takashima, Mitsuo Suga, Toshiaki Suzuki, Yoshikazu Nemoto, Osamu Takai

**Affiliations:** 1 JEOL Ltd., Akishima, Tokyo, Japan; 2 JEOL Technics Ltd., Akishima, Tokyo, Japan; 3 Materials and Surface Engineering Research Institute, Kanto Gakuin University, Odawara, Kanagawa, Japan

**Keywords:** SEM, low vacuum, wet specimen, water droplet, wet cover method, aqua cover

## Abstract

Wet specimens are notoriously difficult to image in scanning electron microscopes (SEM) owing to evaporation from the required vacuum of the specimen chamber. Traditionally, this issue has been addressed by increasing the specimen chamber pressure. Unfortunately, observation under high specimen chamber pressure cannot prevent the initial evaporation effects. The wet cover method, where the original surface water is retained (and, therefore, considered wet), provides a way to introduce and subsequently image specimens that are sensitive to evaporation within a SEM, while preventing evaporation-related damage, and to observe interesting specimen–water interactions.

## Teaser

We developed the wet cover method, which retains original surface water of wet specimens, providing a way to introduce and subsequently image the specimens that are sensitive to evaporation within a SEM, while preventing evaporation-related damage, and to observe interesting specimen-water interactions.

## Introduction

An electron microscope is an indispensable tool for observing small objects. Scanning electron microscopy (SEM) requires the specimen to be in vacuum, since electrons do not propagate in atmosphere or liquid, which is a major disadvantage in case of specimens composed of materials containing relatively low boiling points such as water or other biological fluids, often resulting in the destruction of the specimens. Furthermore, observation of room-temperature liquid water or wet specimens, i.e. materials with a surface layer of water, represents the greatest challenge. One solution is to trap the liquid in an electron transparent cell. Abrams and McBain reported one such environmental cell for transmission electron microscopy (TEM) [1]. Using this structure, observation of a wet specimen was achieved by introducing water vapor from the outside to the specimen [2]. Progress in this technology has been remarkable in recent years owing to the development of micro electron mechanical system (MEMS) technology [3,4]. Another solution is controlling a vacuum environment within the SEM to avoid boiling and reducing the rate of evaporation known as an environmental SEM. Danilatos developed an environmental SEM with a specimen chamber pressure of 6000 Pa or more by using differential pumping, and realizing control of the specimen chamber pressure by introducing various gases including water vapor [5]. Performance of the environmental SEM has been further improved by the development of a new detector [6]. Danilatos also developed an atmospheric pressure scanning electron microscope (ASEM) capable of observing specimen under atmospheric pressure using SEM by optimizing the configuration of the exhaust system and the detector [7]. Electron beam permeable thin films have also been applied to SEM in combination with back-scattered electron detector, and have been widely used not only in biological fields but also in material fields [8–15]. These films have been further improved and used for electron beam excitation assisted optical (EXA) microscopy or the frequency transmission electric-field (FTE) method using SEM; the former converts electron to light and the latter converts electron to electric field [16,17]. The film is used not only for observation but also for forming patterns in a specimen using chemical reactions by electron beam irradiation [18].

Among various methods for observing wet specimens, environmental SEM has been widely used for various types of specimens, including cultured cells, particles in liquid, and water droplets [19], as the surface and cross-section of wet specimens are observed stereoscopically. In addition, environmental SEM has been also used to evaluate the wettability of material surface and behavior of small water droplets, as it observes the surface of water itself [20]. However, researchers reported that observation of water droplet created in the atmosphere is not possible due to evaporation of significant amount of water during initial evacuation, since the water vapor pressure in evacuation becomes significantly low [21,22].

This study examined the usage of wet cover method to observe wet specimens using SEM. The wet cover containing significant amount of water near the specimen prevents evaporation of water from the specimen during initial evacuation. The possibility of increased water vapor pressure in the chamber owing to the evaporation of water vapor from the cover, even after removing the cover, is also indicated. Furthermore, observations of the water droplet created in the atmosphere and the cross-section of wet specimens sensitive to evaporation were made. Possibilities of application field in future were discussed.

## Methods

### Wet cover method

**Fig. 1.**
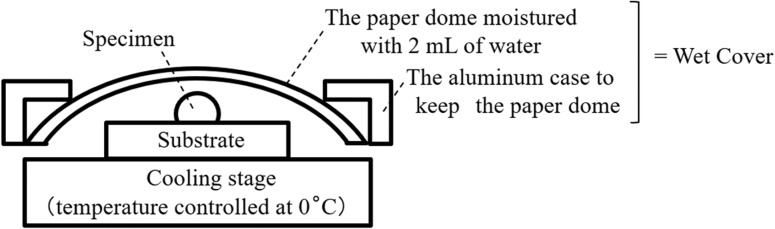
Configuration of wet cover, specimen and the cooling stage. The wet cover is composed of paper containing water and a frame supporting it.

The configuration of the wert cover is described in Fig. 1. The wet cover has an aluminium body with a frame holding a paper with ~2 ml of water. We used KimWipes S-200 (NIPPON PAPER CRECIA CO., LTD., Tokyo) as cover paper. This paper is a 120 mm × 215 mm sheet folded three times; eight layers of paper were used as cover. The cover is dome-shaped and does not completely seal the specimen. Different types of covers with holes on the side (one hole with 3 mm diameter and three holes with 8 mm diameter) were also made to investigate moderate sealing. The distances between the cover and the specimen were also varied.

In the method using this wet cover, the specimen is cooled to 0°C and observed under a pressure environment of 650 Pa. The temperature of the specimen was controlled by a cooling stage using a Peltier device.

**Fig. 2.**
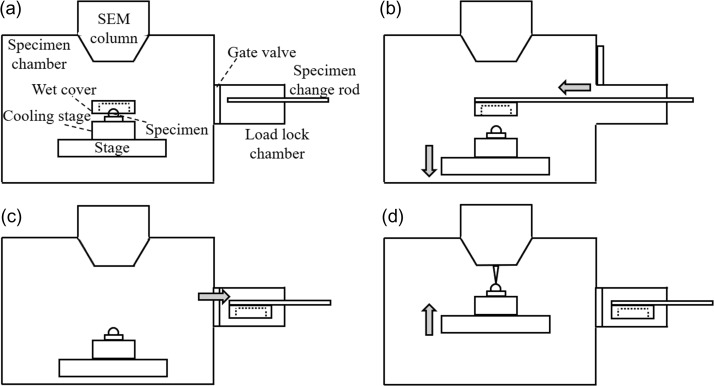
Procedure for installation and removal of the cover, and scanning electron microscopes (SEM) observation. (a) Set the specimen and the wet cover under atmosphere and evacuate to 650 Pa. (b) After stabilizing at 650 Pa, grab the wet cover by the specimen-change rod and lower the stage. (c) Store the wet cover grabbed by the specimen-change rod in the load lock chamber. (d) Raise the stage and start SEM observation.

It is necessary to remove the wet cover for SEM observation. Figure 2 shows the procedure for installation of the wet cover, removal of the cover and SEM observation.

A specimen covered with the wet cover is set under atmosphere and evacuate to 650 Pa (Fig. 2a). When the pressure is stabilized at 650 Pa, the wet cover was grabbed by the specimen change-rod and the stage was lowered (Fig. 2b). After storing the wet cover in the load lock chamber (Fig. 2c), the stage was raised and SEM observation started (Fig. 2d). Note that it takes ~150 s from the start of initial evacuation to removal of the wet cover, and it takes ~120 s from the removal of the wet cover to the start of SEM observation.

In this study, by using the wet cover, we examined how much water can be retained and observed with conventional low vacuum SEM, JSM-IT300 and JSM-IT500 (JEOL Ltd., Tokyo), without externally introducing or controlling water vapor at the time of SEM observation.

### Low vacuum SEM

**Fig. 3.**
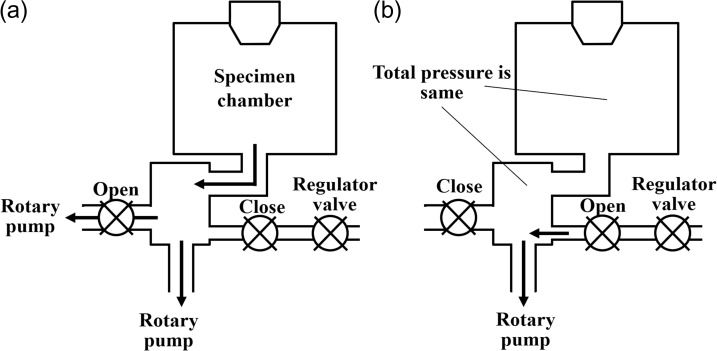
Configuration of the evacuation method for low vacuum SEM. (a) In the initial state of evacuation, two rotary pumps (RP) are used to accelerate evacuation. (b) In the stabilization state, the room connected to the specimen chamber is not only evacuated with one RP but also air is introduced to the same room through the pressure control valve. By balancing evacuation and air introduction, the pressure of the specimen chamber is kept at ~650 Pa.

In low vacuum SEM, the total pressure inside the specimen chamber is kept at 650 Pa maximum as follows. Figure 3 shows the evacuation method of the low vacuum SEM (JSM-IT300 and JSM-IT500) used in this study. Figure 3a shows the method for initial evacuation. The specimen chamber is evacuated with two Rotary pumps (RP) in order to increase the evacuation speed. When the total pressure of the specimen chamber reaches ~700 Pa, the valves are switched for stable evacuation mode (stable pressure) as shown in Fig. 3b. In this case, the chamber is evacuated via next room: the next room is evacuated with one RP and air is introduced via the pressure regulating valve. As a result, the specimen chamber pressure is always kept at ~650 Pa.

### Measurement of water droplet volume change

In order to investigate how the amount of water is kept constant by the wet cover method, changes in outer shape and volume of water droplet made under atmosphere during initial evacuation were measured, with and without the wet cover. These are also measured during the total pressure stabilization time after removal of the wet cover.

As a specimen, 1 μL of water was dropped onto a water-repellent polytetrafluoroethylene (PTFE) sheet (thickness of 100 μm) to make the investigation of the outer shape of droplet easy. After the specimen was cooled to 0°C by Peltier cooling stage, evacuation was carried out from atmospheric pressure to 650 Pa. The water droplet was observed from the lateral direction using an optical camera mounted on the wall of the specimen chamber. With the wet cover, observation was made via a hole of 8 mm in diameter opened in the side of the wet cover. Measurement after removing the wet cover was done separately from these measurements, since the droplet cannot be observed by the camera right after the removal of the cover as described in 2.1. After removing the wet cover, it quickly moved to observable position and observation was carried out. The volume *V* of the water droplet was determined as follows.

**Fig. 4.**
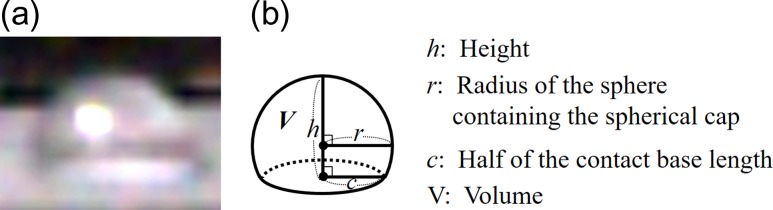
Shape of water droplet and calculation method of volume. (a) Shape of water droplet photographed by optical camera (b) water droplet schematic and parameters for calculating volume

Figure 4 shows water droplet observed with an optical camera, and calculation method of water droplet volume. The volume *V* of the water droplet was obtained from the optical image of Fig. 4a. First, the observed water droplet was approximated as a part of a sphere. The radius is *r*, the height from the contact surface is *h*, and the radius of the contact surface is *c*. Next, on the assumption that the water droplet is rotationally symmetric, we integrated the cross-sectional area of a certain height *x* from height 0 to *h* as shown in equation (1). Since the relationship between *r* and *c* is given by the equation (2), the volume *V* can be obtained by the equation (3).

(1) $$V=\int_{0}^{h}\left\{r^{2}-{\left(x-r\right)}^{2}\right\}\pi dx$$

(2) $$c^{2}=r^{2}-{\left(h-r\right)}^{2}$$

(3) $$V=\frac{\pi }{6}h(3c^{2}+h^{2})$$

### Observation of water droplet and wet specimen by SEM

In order to investigate the amount of water droplet formed under atmospheric pressure that can actually be observed by SEM and the volume of water droplet changes at the time of SEM observation, an optical image of water droplets before evacuation and a change of water droplet at SEM observation were carried out.

On the PTFE sheet (thickness of 100 μm), 1 μl of water was dropped in atmosphere and set it on the Peltier cooling stage. In this case, a specimen stub with the specimen fixing surface being perpendicular to the stage was used to measure the shape and volume of the water droplet by SEM. After the water droplet reached 0°C as photographed using an optical camera under atmosphere, the cover was set. Afterwards, they were evacuated, and the cover was removed as described in Section 2.1. Dynamic SEM observation was carried out to obtain the volume change of the water droplet. The volume of the water droplet was calculated by the method described in Section 2.3. SEM observation was carried out under a pressure of 650 Pa, but in this case scattering of electrons due to gas molecules becomes remarkable. Considering the mean free path, signal amount, and effect of heat, the acceleration voltage was set at 25–30 kV and the irradiation current was set at around 2 μA. The working distance was set to 5–8 mm to minimize scattering. Furthermore, to detect the signal most efficiently with a short working distance, a back-scattered electron detector set just above the specimen was used. SEM images were continuously captured at 1 s/frame and recorded as video. Using the same observation method, the actual specimen was also observed.

One specimen is 1 μl of water dropped on a rose petal (thickness of ~1 mm). The specimen was attached to a specimen stub of aluminum using silver paste to increase the thermal conductivity, and set on a Peltier cooling stage. In this case, a specimen stub processed so that the specimen fixing surface is at 45° to the SEM incident beam was used to observe the interface between water droplet and rose petal. The image capture speed was set to 40 s/frame to obtain clear image.

The second specimen is a potato cross-section. In order to demonstrate the effectiveness of the wet cover method, fractured sections of potato, which is a typical wet specimen, were observed by three methods. The potato was sliced into a piece of 10 mm × 10 mm × 50 mm using a razor. A notch was put near the longitudinal center of the piece and it was cleaved by hand. The opposite side of the cleaved section was scraped off with a knife so that the thickness of the specimen was ~3 mm. The piece was placed on a specimen stub of aluminum with the scraped surface facing downward, and the specimen stub was set on the Peltier cooling stage. After the specimen was cooled to 0°C, observation started using the following method. In method 1, the specimen was evacuated from atmospheric pressure to 650 Pa without the cover and observed with SEM. In method 2, after evacuated from atmospheric pressure to 650 Pa without the cover, water vapor was excessively supplied to condense the surface of the specimen, and SEM observation started. In method 3, after evacuating from atmospheric pressure to 650 Pa using a wet cover method, the cover was removed, and SEM observation started.

### Confirmation that water is not frozen

The specimen is the same as in Section 2.4, and the equipment, procedure, and observation conditions are the same as in Section 2.5. After acquiring the SEM image at the stage temperature of 0°C, the stage temperature was changed to −25°C, and the SEM image was again obtained.

## Results and discussions

### Volume change of water droplet

**Fig. 5.**
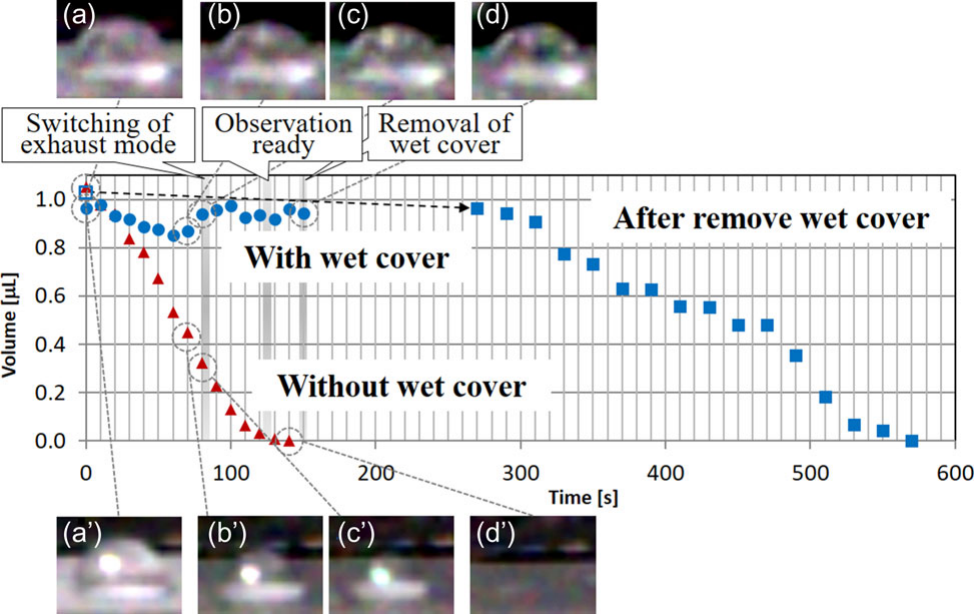
Outer shape and volume of a water droplet. The upper section shows the optical images of the water droplet with the wet cover captured by an optical camera (a–d). The graph in the middle shows the volume of the water droplet during and after evacuation (circle indicates the volume with the wet cover; triangle indicates the volume without the wet cover; closed square indicates the volume after removal of the wet cover; open square indicates the volume before evacuation). The lower section shows the optical images of the water droplet without the wet cover captured by an optical camera (a’–d’). The water droplet on the polytetrafluoroethylene (PTFE) sheet is observed in a cooled state at 0°C.

Figure 5 shows the time dependence of the outer shape and the volume of the water droplet. The water droplet was created in the atmosphere, and evacuation was carried out from atmospheric pressure to 650 Pa.

Without the wet cover (triangles in the graph and a’–d’ in the lower images, Fig. 5), the water droplet gradually became smaller during evacuation, and the volume became 30% of the initial value, when the specimen chamber pressure reached 700 Pa and the evacuation was switched from Fig. 3 (a) to (b) (~85 s after the start of evacuation). The water droplet further became smaller after this switch and disappeared completely ~140 s after the start of the evacuation (average evaporation rate: 7.5 nl/s). Pressure can irradiate the electron beam in ~125 s from evacuation (observation ready), but the water droplet has almost disappeared by that time. The SEM used in this study allows observations 125 s after the start of evacuation, but at this time, almost no water remained.

With water-containing cover, (circles in the graph and a–d in the upper images, Fig. 5), the volume during evacuation was 97 % of the initial value after 150 s from the start of the evacuation, while the volume had been kept within ±12 % of the initial value at this time. After removal of the wet cover (squares in Fig. 5), optical observation using the camera becomes possible 270 s from the start of evacuation. Even at this time, the volume of the water droplet was 0.96 μl, 93% of the initial value of 1.03 μl. The water droplet further became smaller with time (c–d in upper images, Fig. 5), and completely disappeared 570 s after the start of evacuation, i.e. around 300 s after the start of optical observation (average evaporation rate: 7.5 nl/s). This average evaporation rate was about half of the value of that without the wet cover (compare closed squares and triangles in Fig. 5).

### SEM observation of water droplet volume change

**Fig. 6.**
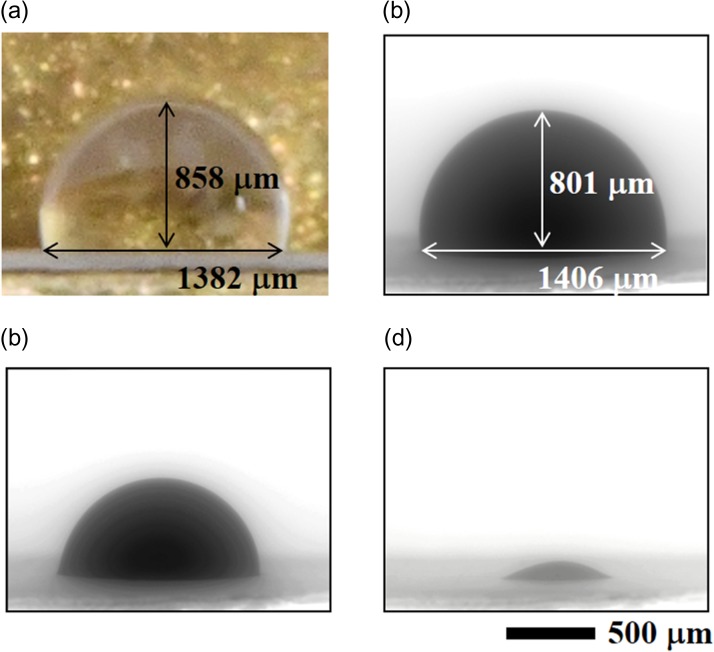
Images of a water droplet created in atmosphere. (a) Optical image taken under the atmosphere. (b–d) SEM images of the water droplet observed under pressure of 650 Pa. (b) Immediately after the start of SEM observation. (c) 80 s after (b); (d) 170 s after (b). The volume of the water droplet calculated from the image in (a) was 0.97 μl, and from (b) was 0.89 μl. The difference of the volumes was <10%. SEM observation conditions. Chamber pressure: 650 Pa, acceleration voltage: 25 kV, irradiation beam current: 2 μA, working distance: 5 mm, back-scattered electron image, 1 s/frame record as video.

Figure 6 shows images of a water droplet made in atmosphere. After acquiring the optical image of water droplet under atmosphere, evacuation was performed to obtain SEM image. This demonstrated that a water droplet created under atmosphere can be observed using SEM. The volume of the water droplet calculated from the SEM image of Fig. 6 (b) and the volume of the water droplet calculated from the optical image of Fig. 6 (a) were very close (a difference smaller than 10 %). It indicates that the wet cover method is applicable for SEM observation of most specimens sensitive to evaporation, except for the cases where precise measurements of water droplet are needed.

The water droplet decreased with time (Fig. 6(c–d)), and the average evaporation rate was 4.9 nl/s, since a water droplet of 0.89 μl could be observed for ~180 s. This value is 1.5 times greater as compared with the value of 3.2 nl/s for optical observation. The reason of the difference would be the influence of heat due to electron beam irradiation.

Although caution must be taken because the observable time is limited by the influence of evaporation, it is clearly established that the wet cover method is applicable for SEM observation of a droplet created in atmosphere using conventional low vacuum SEM.

### SEM observation of water droplet on rose petal using wet cover method

**Fig. 7.**
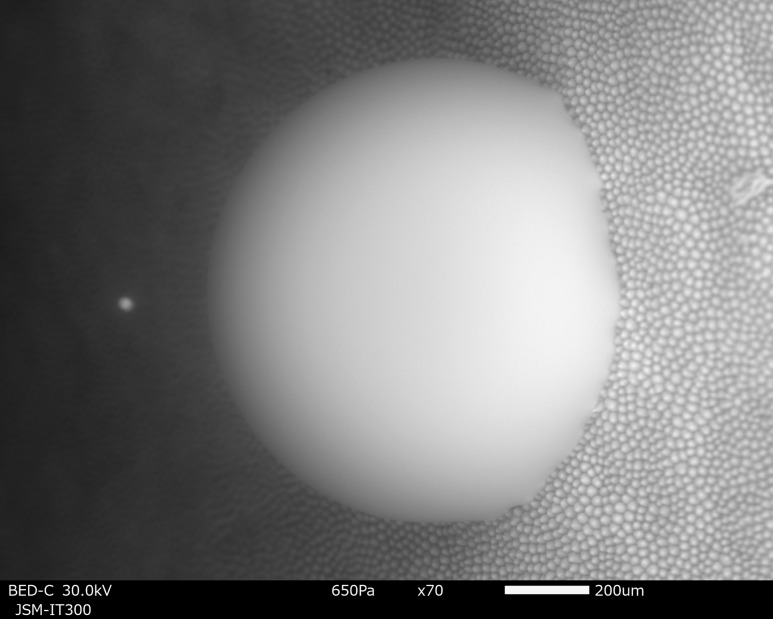
SEM image of a water droplet of 1 μl on a rose petal. The droplet was created in atmosphere. The wet cover method was used. SEM observation conditions. Chamber pressure: 650 Pa, acceleration voltage: 25 kV, irradiation beam current: 1.8 μA, working distance: 8 mm, back-scattered electron image, 40 s/frame.

Rose petals have a small uneven structure on the surface, and it is known that this structure has a water-repellent effect [23]. Figure 7 shows SEM image of a water droplet dropped on a rose petal observed by the wet cover method. One microlitre of water was dropped on the petal in the atmosphere and it was maintained during evacuation using the wet cover.

Both the fine structure of the rose petal surface such as roughness and the shape of the water droplet in contact with the rough petal surface have been clearly observed. It is well known that water repellence is strongly dependent on fine structures of a surface, and this method would be effective for investigating these relationships in the future. Environmental SEM has been widely used to investigate the relationship between structure and water repellence [20]. However, with environmental SEM, water droplets have to be generated inside the SEM chamber by condensation of supersaturated water vapor introduced into the chamber after initial evacuation, since water droplets fabricated in atmosphere evaporate during the initial evacuation [5,20,22,24,25]. In this case, the size of the droplets is limited. It is impossible to create water droplets in the order of millimeters as shown in Fig. 10. The air pockets between the surface with fine structure and the water droplets is important for water repellence. Jung reported that the air pockets disappeared, and transition of the state occurred during the shrinkage of the droplet from mm size to sub mm size due to evaporation [20]. For this reason, observation of the evaporation process for mm size water droplet is important in future.

### Comparison of observation methods for a potato cross-section

**Fig. 8.**
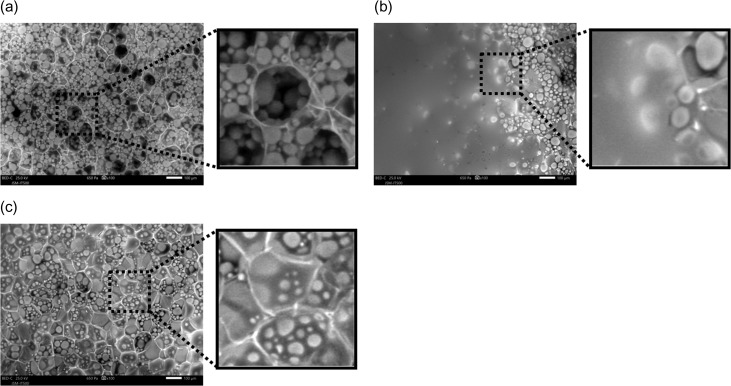
SEM images of cross sections for potatoes observed by different methods. (a) Without the wet cover. (b) Supplying excess water vapor after initial evacuation without the wet cover. (c) With the wet cover. SEM observation conditions. Chamber pressure: 650 Pa, acceleration voltage: 25 kV, irradiation beam current: 2 μA, working distance: 5 mm, back-scattered electron image, 10 s/frame.

Figure 8 shows the results of SEM observations for the cross sections of potatoes by three different methods.

Using Method 1 (SEM observation without the wet cover method), no water remained between the starch granules, and they adhered to the cell wall (Fig. 8(a)). In addition, the cells shrank due to drying and the starch granules spilled out of the cells.

Using Method 2 (SEM observation with excess water vapor supplied after initial evacuation without the wet cover), water did not remain between the starch granules to make them visible, and once dried, the cells shrank and the starch granules flowed out of the cells (Fig. 8(b)). In addition, starch granules on the surface of the specimen became invisible, since the surface was covered with water derived from the excessive supply of water vapor.

Using Method 3 (SEM observation using the wet cover method), water remained between the starch granules and the starch granules were retained in the cells because there was no shrinkage (Fig. 8 (c)). As a result, the starch granules floating in the cells were observed.

The water content of a potato is high, around 80% of the total mass. In the SEM images using Method 1 and Method 2, the starch granules overflowed from the cells and the amount of starch granules contained in the cells could not be measured. On the contrary, using Method 3, the wet cover method, the starch granules originally distributed in the cells were visible and could be counted. The number of starch granules contained in a potato is not only related to taste but also important in the field of starch production, necessitating studies on the control of the number and size of starch granules in the cells [26]. Therefore, the wet cover method, which allows rapid observation for the appearance of the starch granules in natural state, would be beneficial in this field.

### Confirmation that water is not frozen

**Fig. 9.**
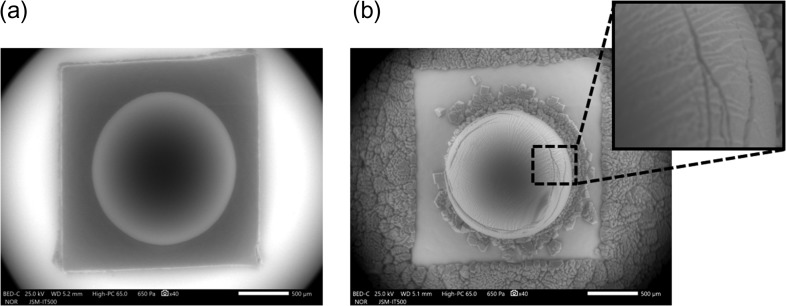
Temperature dependence of SEM images of a water droplet using a wet cover method. (a) The water droplet observed at 0°C. (b) The water droplet observed at −25°C. In (a), liquid state aspect was observed, but in (b) fringes and cracks were observed on the surface, showing it was frozen. SEM observation conditions. Chamber pressure: 650 Pa, acceleration voltage: 25 kV, irradiation beam current: 2 μA, working distance: 5 mm, back-scattered electron image, 1 s/frame record as video.

Figure 9 shows the temperature dependence of the SEM image of a water droplet observed at 0°C (a) and at −25°C (b). Figure 9(a) shows the surface of the droplet is smooth similar to the experiment results in Section 3.2. On the contrary, Fig. 9(b) shows fringes and cracks in the surface of the droplet.

This indicates that the droplet in fact actually frozen, while the observed temperature of at −25°C corresponds to the temperature for solid phase of a water at 650 Pa. From the comparison of the surfaces in Fig. 9 (a) and (b), it was confirmed that the water droplet observed at 0°C was in the liquid phase.

### Conditions for observing water and wet specimens by SEM

Observation of wet specimens with SEM requires the control of three parameters:

1. Total pressure inside the SEM chamber.
2. Temperature of the specimen.
3. Partial water vapor pressure limit.

First, total pressure inside the SEM chamber should be controlled to prevent boiling of water. The surface morphology of water changes due to boiling, possibly deforming the material around the water. It is necessary to keep the total pressure inside the specimen chamber above the saturated water vapor pressure during both evacuation and SEM observation, since the water boils when the total pressure of the gas surrounding the specimen falls below the saturated water vapor pressure [27,28].

**Fig. 10.**
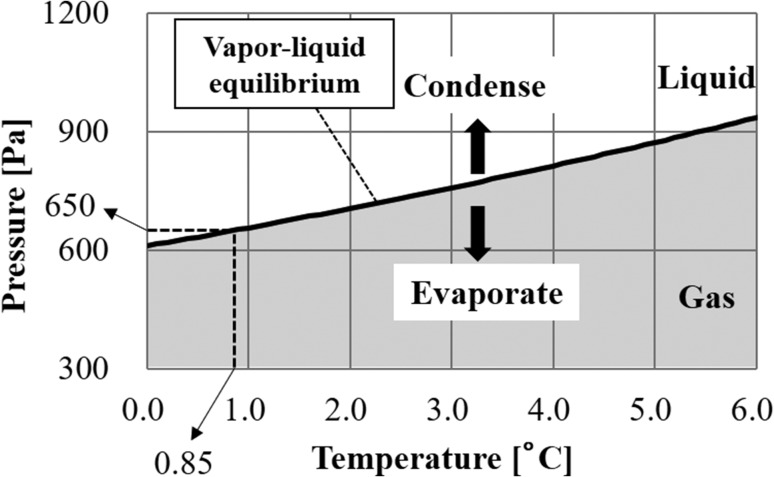
Saturated water vapor pressure curve. When the environment of water and gas are mixed, water boils when the total pressure of the gas becomes lower than the saturated water vapor pressure. If the partial water vapor pressure of the gas is higher than the saturated water vapor pressure, the gas will condense and if it is lower the water evaporates. In order to keep the amount of water constant, it is necessary to keep the water vapor pressure of the gas around the saturated water vapor pressure.

Second, the temperature of the specimen should be controlled. Since the maximum value of the total pressure is 650 Pa in the conventional low vacuum SEM, it is necessary to search for a temperature at which the saturated water vapor pressure becomes <650 Pa. This happens when the temperature is 0.85°C or less as shown in the saturated water vapor pressure curve [29] of Fig. 10. During SEM observation, if the total pressure is high, scattering of electrons increases, and the image becomes unclear. In order to reduce scattering of electrons due to gas, it is desirable to maintain the specimen temperature in the vicinity of 0°C, which is the lowest saturated water vapor pressure while maintaining the liquid phase.

Another condition for wet specimen observation is the partial water vapor pressure limit [30]. In order to maintain the amount of water in wet specimens, it is necessary to set the partial water vapor pressure in the gas near the specimen at around the saturated water vapor pressure [27,28]. This is because condensation occurs when the partial water vapor pressure is higher than the saturated water vapor pressure and evaporates when the partial water vapor pressure is low.

Partial vapor pressure was estimated for conventional low vacuum SEM, since it does not control water vapor pressure. Temperature of air outside of SEM and relative humidity (water vapor partial pressure/saturated water vapor pressure × 100%) are assumed to be 25°C and 50%. In this case, the saturated water vapor pressure of the air is 3175 Pa and the partial water vapor pressure is 1588 Pa. The partial water vapor pressure is calculated to be 10.2 Pa, when the total pressure is evacuated to 650 Pa assuming the ratio of the partial water vapor pressure to the total pressure remains the same. That is the water vapor pressure during evacuation in the low vacuum SEM is between 1588 Pa and 10.2 Pa, and the pressure at the time of SEM observation is particularly ~10.2 Pa, when water vapor is not controlled. It is necessary to increase the partial water vapor pressure during initial evacuation and SEM observation, and the effect of the cover is discussed in the next section.

### Effects of wet cover method

**Fig. 11.**
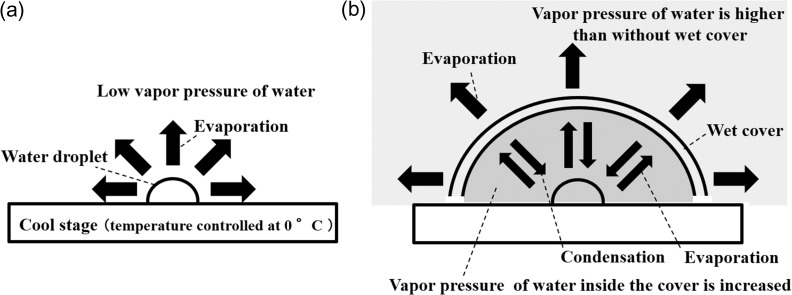
Configuration of the effect of the wet cover. (a) Without the wet cover. Water in specimen evaporates when the water vapor pressure of the specimen chamber is low. (b) With the wet cover. Amount of water in specimen is kept almost constant by balancing the amount of evaporation and condensation from the specimen. The water vapor pressure inside the specimen chamber increases by the evaporation from the cover.

Figure 11 shows the effect of wet cover. Without wet cover (Fig. 11(a)), the water vapor pressure in the specimen chamber is calculated to be from 1588 Pa to 10.2 Pa through the evacuation process, when temperature and relative humidity of outside air are assumed to be 25°C and 50%, respectively, as described above. During this time, the water vapor pressure decreases exponentially, and from the start of the evacuation, it becomes less than half of the initial value after 10 s and 20 Pa after 30 s. Therefore, the water droplet evaporates during the initial evacuation. With wet cover (Fig. 11(b)), during evacuation, the water vapor pressure in the specimen chamber and near the specimen decreases, but water contained in the cover evaporates, and increases the water vapor pressure near the specimen. If these are balanced, the water content of the specimen will be kept constant during evacuation.

In addition, the wet cover increases water vapor pressure not only in the vicinity of the specimen but also in the entire specimen chamber, suggesting that the water vapor pressure in the specimen chamber becomes close to the saturated water vapor pressure when the specimen chamber is not evacuated. In the SEM used in this study, the gas in the specimen chamber is slightly evacuated, but the amount of the evacuation is limited after stabilization of pressure, as described in Section 2.2 (Fig. 3 (b)). Therefore, the water vapor pressure in the specimen chamber is close to the saturated water vapor pressure with wet cover, and the pressure would be kept high at some extent after the removal of the cover since the amount of evacuation is restricted. In fact, from the result in Section 3.1, the average evaporation rate after removal of wet cover is less than half of the value without the cover, indicating that the water vapor pressure after wet cover removal is higher than that without wet cover method. Since the water vapor pressure inside the specimen chamber gradually decreases mainly due to diffusion of water vapor, the time available for SEM observation is limited after removing the cover.

In some cases, it is effective that the water of the specimen surface is gradually evaporated. Stokes *et al.* pointed out that target object buried in the aqueous solution became visible by slow evaporation of water during observation when they observed mammalian cells using environmental SEM [31]. To achieve slow evaporation during observation, it is necessary to lower the water vapor pressure in the specimen chamber below the saturated water vapor pressure. The wet cover method is especially suitable for exposing the target object, since water vapor pressure in the specimen chamber is lower than saturated water vapor pressure during SEM observation, allowing slow evaporation of water pointed out by Stokes *et al.* For specimens such as cultured cells, significant amounts of aqueous solution covering the cells can be added in atmosphere before evacuation, retaining water even by strong evaporation of environmental SEM during initial evacuation. However, for specimens with limited water content such as the cross-section of potatoes, control of evaporation during initial evacuation is crucial. Therefore, the wet cover method, allowing control of evaporation during initial evacuation, is valuable for various specimens sensitive to evaporation. It should be noted that for cultured cells, Stokes *et al.* also showed that SEM observation was achieved with a specimen chamber pressure 270–400 Pa lower than the saturated water vapor pressure at temperature between 2°C and 6°C (~700–940 Pa) typically used in environmental SEM, since the vapor pressure of aqueous solutions containing macromolecules and ions is lower than that of pure water. For similar specimens, the same effect is expected with the wet cover method.

In addition, the following examination was made to investigate whether the shape and water content of the cover is optimum. The existence of the hole or holes on the side of the cover and the distance between the cover and the specimen did not alter the results of evaporation. These results show that evaporation is not sensitive to the detailed shape of the cover. On the other hand, the presence of the gap between the cover and the specimen is important to maintain the shape of the surface of the specimen. Water absorbency of the paper in the cover is also important since water should be uniformly present on it. The water content of ~2 ml was typically included in the cover. If the volume was too high, the specimen got wet, and if it was too small, the specimen dried in the initial evacuation.

### Comparisons with other microscopes

Environmental SEM allows observation of surfaces in wet specimens, i.e. surface exposed to air from water or surface of water itself. However, as discussed above, environmental SEM is hard to apply in specimens sensitive to evaporation. The wet cover method allowed for observation of wet specimens.

TEM with an environmental cell and SEM with a wet capsule have been widely used to observe wet specimens. They succeeded in observations of structures in liquid including magnetic materials in bacteria or organelles in cultured cells [32]. In addition, they can be used to observe specimens sensitive to evaporation, since the environmental cell and wet capsule have semi-closed structures. However, they cannot observe surfaces, either surfaces exposed to air from water or surfaces of water itself.

Air SEM and similar microscopy could be used to observe the surfaces [14,15], but the image quality is degraded due to strong scattering of primary electrons by air. To improve image quality, distance between thin film and a specimen should be close, usually <100 μm, which restricts the roughness size of a specimen surface. Therefore, it is difficult to stereoscopically observe a water droplet of the 1 mm order. The efforts to recover the image quality by calculation has been made [15].

Optical microscopy is a valuable method to observe the surface of water droplet, however, its spatial resolution is limited by the wavelength of light. Scanning probe microscopy (SPM) and super-resolution optical microscopy (SR) are also effective methods for observing those smaller than the wavelength of light. SPM has been actively applied to the observation of objects in liquid in recent years, and it has been applied to dynamic observation of proteins [33]. However, it cannot be applied to the observation of the liquid surface itself. SR is also actively utilized for specimen observation in the field of life sciences, and those with position identification accuracy of 20–30 nm are also in practical use [34]. However, these are also not able to observe the surface itself of the liquid. By using the wet cover method, it is possible to observe the relationship between the appearance of water itself and the fine surface structure in detail.

## Concluding remarks

Modification of a conventional SEM has produced a method of imaging wet specimens sensitive to evaporation stereoscopically. The wet cover method achieved rapid observation of wet specimens sensitive to water evaporation, as well as of water droplets itself formed under atmosphere. This method could be applied to observe various evaporation sensitive specimens, water droplets for studies of repellence, and the evaporation process itself for evaporable specimens.
